# The Journal and the Colleges

**Published:** 1893-06

**Authors:** 


### *EDITORIAL.*

---

The office of THE JOURNAL is in room 330 Equitable Building.

Address communications relating to the Editorial Department to DR. L. B. GRANDY, Atlanta, Ga., Box 431.

Business communications should be addressed to DR. M. B. HUTCHINS, Atlanta, Ga., Box 431.

All remittances should be made payable to "ATLANTA MEDICAL AND SURGICAL JOURNAL."

Articles for publication should reach this office not later than the 15th instant.

The editors are not responsible for views expressed by contributors.

---

### THE JOURNAL AND THE COLLEGES.

---

We have announced before in this place that the attitude of this JOURNAL with respect to the medical colleges of this city shall be one of absolute neutrality. We recognize that both colleges may be our friends, and that it would be a clear disadvantage for THE JOURNAL to be the organ of either.

These remarks are called out again from the fact that an article by the managing editor appeared in our May issue containing an allusion to one of the colleges which has been thought to be both severe and undeserved. However that may be, the paragraph expressed the opinion of an individual only, *not* THE JOURNAL'S. An announcement stands at the top of this page that the editors and proprietors do not hold themselves responsible for views expressed by contributors. This rule applies also whenever either of them contributes an "original communication" to these pages. The editorial pages will reflect the sentiment of THE JOURNAL, and while the proprietors will endeavor to maintain their position of neutrality, and while they will refuse to cast its influence for or against either school, they reserve the privilege of honest criticism whenever comment seems appropriate. The freedom of independent journalism at least gives us this liberty.

